# Hand and wrist osteotomies in Sweden: a population-based study of 6911 interventions from 2008 to 2023

**DOI:** 10.1177/17531934261417178

**Published:** 2026-02-11

**Authors:** Viktor Mili-Schmidt, Mats Wadsten, Elsa Pihl, Cecilia Mellstrand Navarro, Michael Axenhus

**Affiliations:** 1 Department of Orthopaedic Surgery, Danderyd Hospital, Sweden; 2 Department of Clinical Sciences at Danderyd Hospital, Karolinska Institutet, Sweden; 3 Department of Surgical and Perioperative Sciences, Umeå University, Sweden; 4 Department of Clinical Research and Education Södersjukhuset, Karolinska Institutet, Sweden

**Keywords:** Corrective osteotomy, epidemiology, hand, hand surgery, National Patient Register, osteotomy, shortening osteotomy, Sweden, trends, wrist

## Abstract

**Introduction:**

Elective osteotomy is a well-established treatment for malunited fractures and degenerative deformities of the wrist and hand. However, contemporary population-based evidence regarding temporal, demographic and regional trends is limited.

**Methods:**

This nationwide observational study analysed all wrist and hand osteotomies in Sweden between 2008 and 2023 using data from the National Patient Register. Procedures were identified by NOMESCO codes for rotational/angular osteotomy (NDK59, NCK59) and lengthening/shortening osteotomy (NDK69, NCK69). Incidence rates were calculated per 100,000 inhabitants and stratified by sex, age and region. Predictive modelling was applied to estimate trends up to 2040.

**Results:**

A total of 6911 wrist and hand osteotomies were identified between 2008 and 2023, including rotational/angular osteotomies (NDK59: 2833; NCK59: 1276) and lengthening/shortening osteotomies (NDK69: 1682; NCK69: 1120). The annual incidence declined steadily across the study period. Women consistently accounted for more procedures than men across all osteotomy types. Incidence was highest among middle-aged and older adults, with younger patients representing a small proportion of cases. Substantial regional variation was observed throughout Sweden, with some regions carrying out several times more procedures than others. Forecast modelling predicts a continued decline to 2040.

**Conclusions:**

The incidence of osteotomies of the wrist and hand in Sweden has declined markedly over the past 16 years, which may reflect improvements in primary fracture care. These findings reflect evolving surgical practices, demographic influences and systemic factors such as regional disparities. A continued decline in osteotomy procedures has major implications: as case volumes decrease, exposure for trainees diminishes, with fewer surgeons proficient in osteotomies, even though patients will continue to benefit from the procedure. Similar concerns have been raised in the context of other high-skill orthopaedic interventions. This might prompt centralization of complex cases and cross-border collaborations to ensure adequate surgical competence.

**Level of evidence:**

III

## Introduction

Osteotomy remains an important intervention for the treatment of post-traumatic malunions and degenerative deformities of the wrist and hand when conservative therapy is insufficient. Population-based data suggest that the overall frequency of these procedures is low. A Finnish nationwide registry study reported a relatively low incidence (1 %) of corrective osteotomy after distal radial fractures (DRFs) treated non-operatively (Raudasoja et al., 2024). Similarly, analyses of Medicare-aged populations in the United States found that patients treated conservatively are more likely to require later osteotomy than those undergoing primary fixation, with advanced age identified as a significant risk factor (Dineen et al., 2019).

Long-term outcomes of corrective osteotomy support its clinical utility, with patients reporting sustained improvements in alignment, function and pain relief years after surgery (Andreasson et al., 2020). Technical innovations have further refined these procedures: the multiple drill-hole osteotomy technique has shown high accuracy in the correction of phalangeal and metacarpal deformities, and 3D-planned patient-specific guides have been introduced to improve precision and reduce complications in complex malunions (Haider et al., 2017; Vlachopoulos et al., 2016).

Osteotomy is also an option in the management of ulnar impaction syndrome, where ulnar shortening osteotomy has produced durable improvements in pain and function over a 10 year follow-up (Mesas Aranda et al., 2024). Nevertheless, there is an increasing role for alternative interventions, including arthroscopic wafer procedures and prosthetics, highlighting developments in hand and wrist surgery (Oh et al., 2018).

There are knowledge gaps in the national-level use of wrist and hand osteotomies. A few studies provide some data on temporal, demographic or regional trends (Andreasson et al., 2020; Haider et al., 2017; Raudasoja et al., 2024). This study aimed to map the current and future trends of osteotomy in Sweden by examining the incidence of rotational/angular (NDK59) and lengthening/shortening (NDK69) osteotomies from 2008 to 2023 and predicting future trends up to 2040.

## Methods

### Study design and setting

This observational, population-based registry study is based on open-source surgical data derived from the National Patient Register (NPR) from 2008 to 2022. The NPR is a validated data base often used for retrospective observational studies (Ludvigsson et al., 2011). The study followed the RECORD guidelines (Benchimol et al., 2015).

### Setting

The Swedish National Health Service provides universal healthcare for all Swedish citizens, including free emergency treatment, general hospital care and outpatient services. Swedish residents are assigned a unique and permanent personal identification number, which remains with them until death or emigration. This number is used for all interactions with public and private healthcare and is integral to national healthcare registers.

### Data source

The NPR is a nationwide registry containing detailed information on patients treated within the Swedish healthcare system, including hospitalizations and outpatient care. Inpatient data has been included since 1964 (nationwide since 1987) and specialized outpatient care was added in 2001. Until 2021, the registry was updated annually (Swedish National Board of Health and Welfare. National Patient Register, n.d.). Since June 2021, it has been updated monthly, incorporating late or corrected data even after publication. The NPR includes comprehensive details on surgical procedures, such as geographical distribution, age and sex. Diagnosis codes are based on the International Classification of Diseases (ICD-10) since 1994, and surgical procedures follow the NOMESCO classification system (ICD-10, 2011; Nomesco, 2011 NOMESCO Classification of Surgical Procedures, version 1.16). All hospitals in Sweden, both public and private, are required to report data.

### Patients

This study included all individuals aged 15 years or older at the time of surgery with residency in Sweden who underwent an osteotomy in the hand or wrist between 1 January 2008 and 31 December 2023. Only individuals with a Swedish personal identification number were included. All osteotomy surgeries in the hand were identified in the NPR using NOMESCO surgical procedure codes for corrective osteotomy (codes: NDK59, NDK69, NCK59 and NCK69).

### Ethics

The data used in this study are publicly available and anonymized. Therefore, ethical approval and informed consent were not sought. Clinical trial number is not applicable.

### Statistical analysis

The annual incidence of each procedure was calculated per 100,000 inhabitants to account for population changes. Data were stratified by surgical method, sex, age group and region to facilitate trend comparisons over time. Incidence rates were determined by dividing the number of patients by the total population, as reported by Statistics Sweden (2025, https://www.scb.se/en/). Significant differences between groups were assessed using Student’s *t*-test. To predict future trends, regression analysis was used to fit exponential, linear, logarithmic, second-order polynomial and power regression models for each incidence trend. Predictive analysis was based on the best-fitting model according to *R*^2^ value, with 95% confidence intervals applied where relevant. A two-sided *p*-value of <0.05 was considered statistically significant.

## Results

A total of 6911 wrist and hand osteotomies were identified between 2008 and 2023, including rotational/angular osteotomies (NDK59: 2833; NCK59: 1276) and lengthening/shortening osteotomies (NDK69: 1682; NCK69: 1120).

For rotational/angular procedures, women consistently underwent more operations than men. Similarly, for lengthening/shortening procedures, women underwent more operations than men (Table 1).

**Table 1.** Annual number of osteotomy operation (rotational/angular and lengthening/shortening) in Sweden between 2008 and 2023, stratified by sex.

Type of osteotomy	Sex	2008	2009	2010	2011	2012	2013	2014	2015	2016	2017	2018	2019	2020	2021	2022	2023
Angle or rotation	Men	3.1	2.8	2.5	2.8	2.8	2.6	2.9	2.4	2.4	2.2	2.1	2.2	1.5	2.1	2.0	1.8
Angle or rotation	Women	3.4	3.4	3.7	4.0	4.2	4.0	4.8	4.0	4.5	3.8	3.9	4.8	3.1	3.7	4.1	3.1
Shortening or lengthening	Men	1.7	1.7	1.5	1.7	1.6	1.9	1.8	2.0	1.5	1.4	1.3	1.7	1.1	1.2	1.0	1.4
Shortening or lengthening	Women	2.9	3.1	3.1	3.4	3.0	2.9	3.0	2.3	3.0	2.7	2.0	2.7	2.1	2.0	2.8	2.9

**Figure 1.**
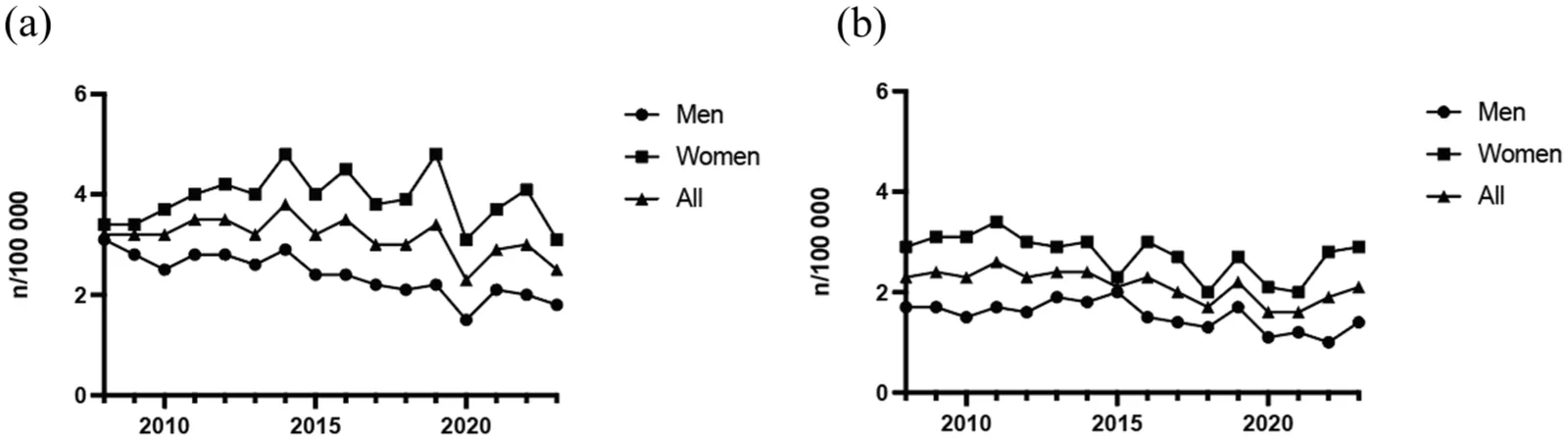
Annual incidence rates of osteotomy in the hand and wrist: (a) rotational/angular; and (b) lengthening/shortening) stratified by sex (2008–2023).

The was a gradual decline in rotational/angular osteotomies in men and women, with women showing higher rates. Lengthening/shortening osteotomies followed a similar declining trend, with men showing consistently lower rates than women (Figure 1).

**Figure 2.**
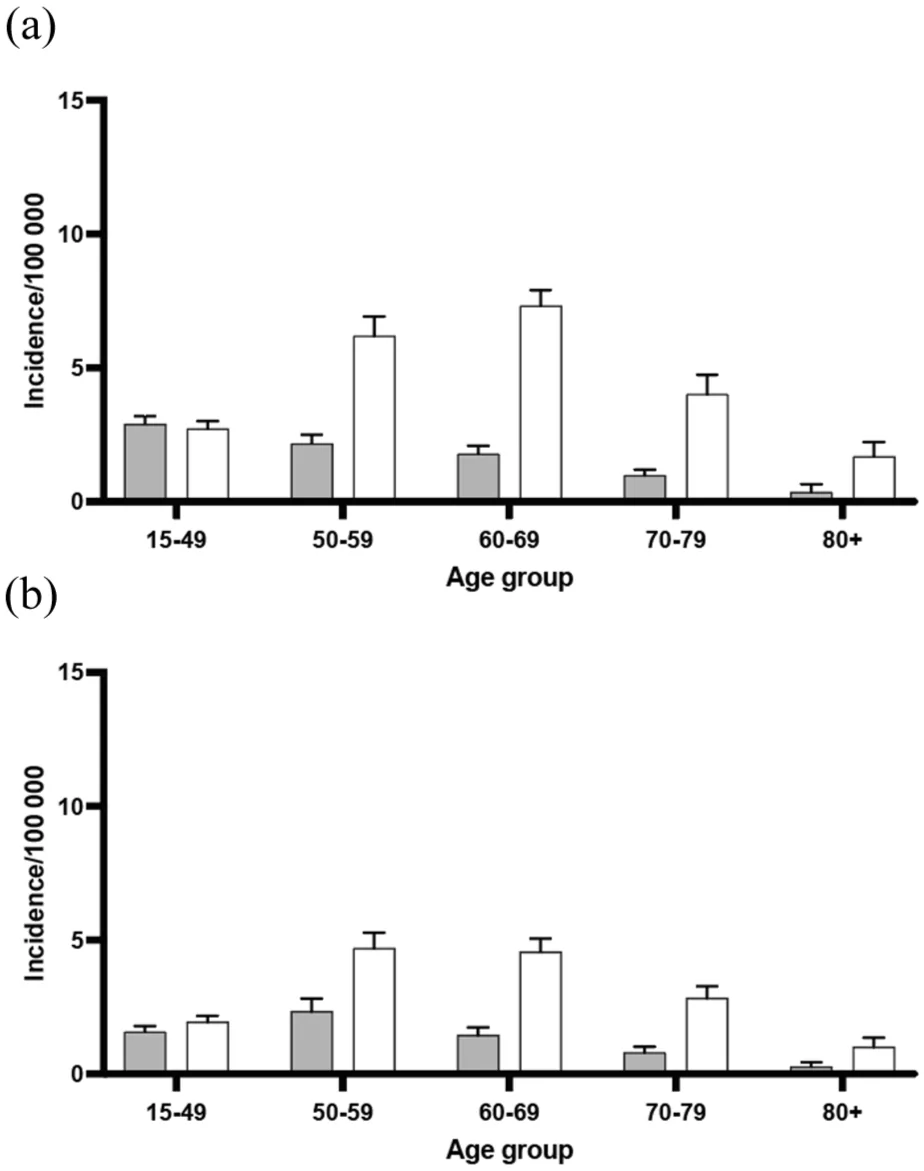
Age group distribution of patients undergoing osteotomy in the hand and wrist (A: rotational/angular; B: lengthening/shortening) over the study period. White indicates women and grey indicates men.

There was a concentration of interventions among middle-aged and older adults. Both rotational/angular osteotomies and lengthening/shortening osteotomies were less frequently done in younger age groups, with incidence rates increasing steadily in middle-aged individuals before tapering off in the elderly population (Figure 2).

**Figure 3.**
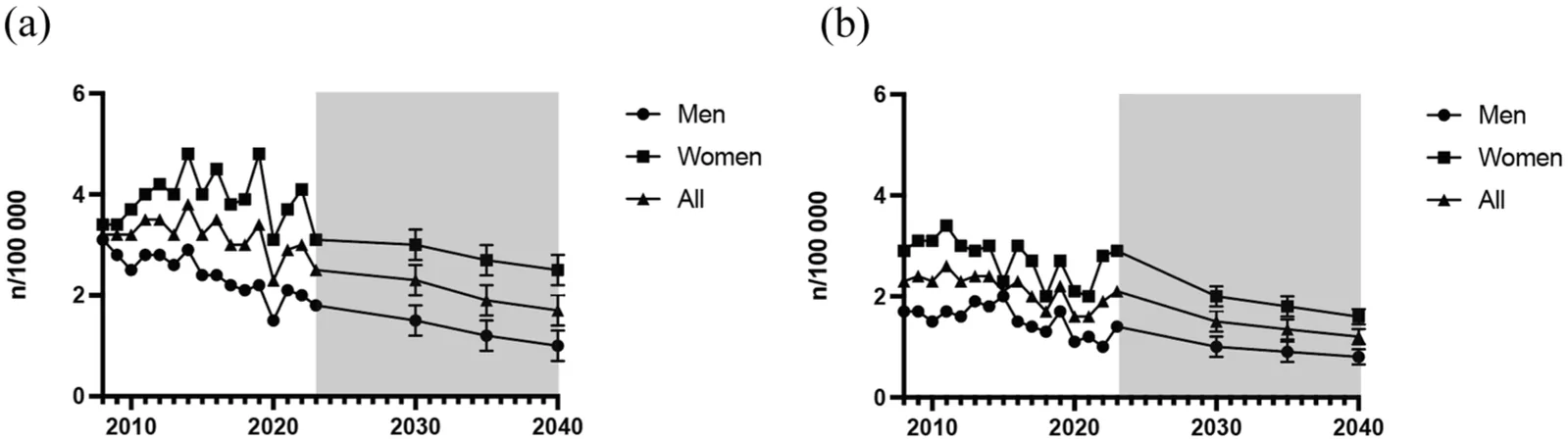
Predicted incidence trends of osteotomy procedures in the hand and wrist: (a, rotational/angular and b, lengthening/shortening) up to 2040. Grey background indicates predicted time-period.

Using regression models, future trends in incidence rates for rotational/angular osteotomies and lengthening/shortening osteotomies were predicted. Rotational/angular osteotomy is expected to decline further, reaching minimal levels by 2040 (Figure 3a), while lengthening/shortening osteotomy shows a similar decline but at slightly higher levels (Figure 3b).

**Figure 4.**
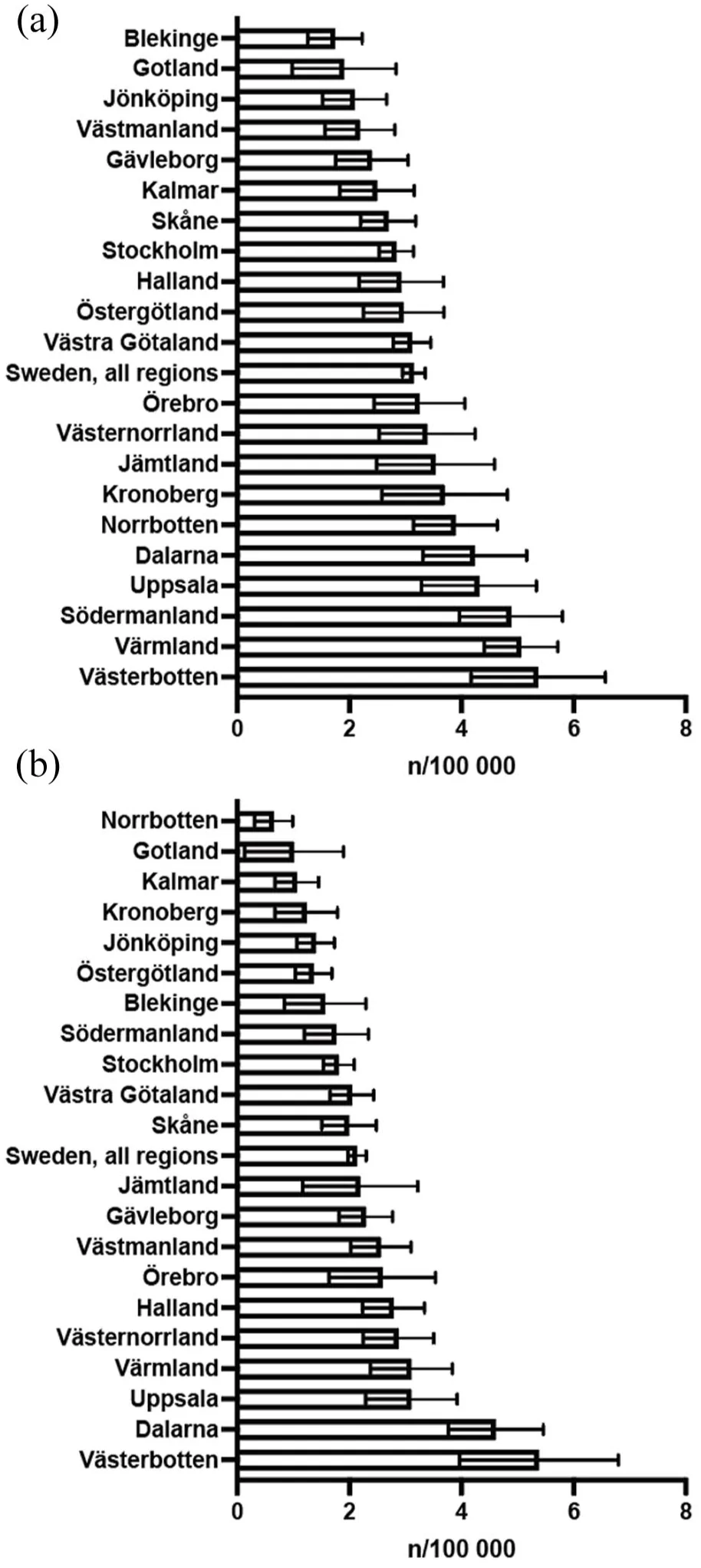
Regional variation in incidence rates per 100,000 inhabitants of corrective osteotomy in the hand: angular/rotational (a) and lengthening/shortening (b) over the study period (2008–2023).

**Figure 5.**
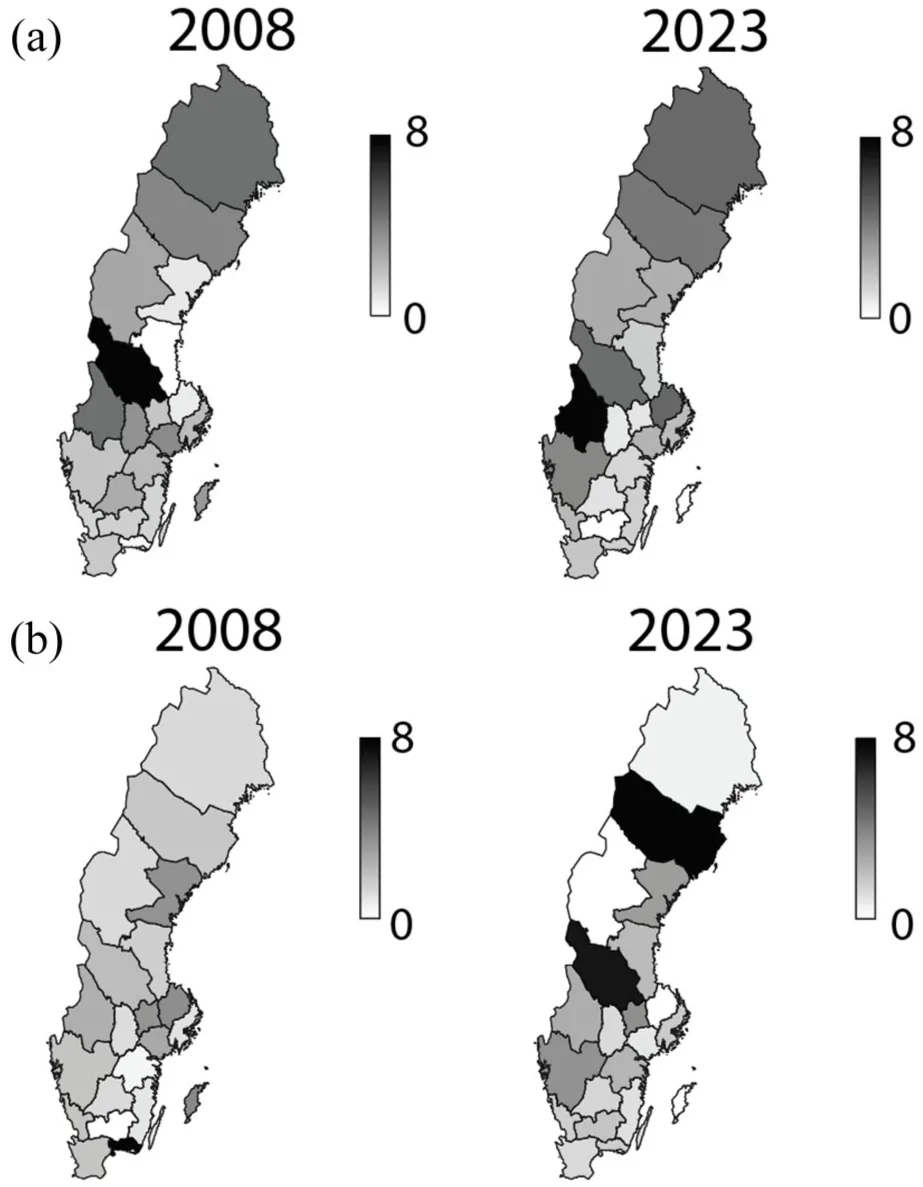
Regional variation in incidence rates per 100,000 inhabitants of corrective osteotomy in the hand: angular/rotational (a) and lengthening/shortening (b) during 2008 and 2023.

Mean regional incidence of osteotomy varied significantly during the study period (Figure 4). Rotational/angular osteotomy was most frequently done in southern Sweden (Figures 4(a) and 5(a)) and had a slightly higher incidence than lengthening/shortening osteotomy (Figure 3).

Regional differences were evident in the incidence rates of rotational/angular osteotomy (Figures 4(a) and 5(a)) and lengthening/shortening osteotomy (Figures 4(b) and 5(b)). Certain regions had consistently higher rates, while others maintained significantly lower rates over time.

## Discussion

This population-based study provides insights into long-term trends in osteotomy procedures of the wrist and hand in Sweden. Over a 16-year period, a decline in both rotational/angular osteotomies (NDK59, NCK59) and lengthening/shortening osteotomies (NDK69, NCK69) was observed. Importantly, these reductions were consistent across most demographic strata, although there were sex- and region-specific differences. Predictive modelling indicates that there will be a further decline until 2040, raising questions about both the changing epidemiology of wrist and hand pathology and the future role of hand and wrist osteotomy in surgical practice.

Changes in the number of osteotomies over time may be influenced by several factors. Better early fracture treatment may reduce the need for later corrective surgery, whereas congenital or acquired deformities may still require an osteotomy. New surgical techniques may affect the development in both directions by offering alternative treatments or creating new indications. In addition, available resources and access to specialists can also influence the incidence of these procedures. Accordingly, the observed reductions should not be interpreted as evidence that fewer patients ‘should’ receive osteotomy; rather, they indicate that osteotomy is being performed less frequently, and the clinical reasons for this shift require evidence from complementary data sources.

Previous studies have reported distal radial osteotomy rates around 0.5–1% (Dineen et al., 2019; Raudasoja et al., 2020). In contrast, the present study describes the incidence over time and reveals a declining trend. Primary fixation of DRFs is increasing, especially in the active elderly group in Sweden, which may explain the sharp decline observed in Sweden (Mellstrand-Navarro et al., 2014; Wilcke et al., 2013). Future multinational comparisons are warranted to determine whether surgical treatment of DRFs in the active elderly is associated with true decreases in the requirement for osteotomy. Moreover, the decline observed in Sweden may also reflect evidence-based conservative management of primary metacarpal fractures which carry a lower risk of rotational deformity compared with primary surgical fixation (Peyronson et al., 2023).

For both rotational/angular and lengthening/shortening osteotomies, there were substantially more procedures in women than men. This may in part be explained by the higher incidence of DRFs in women (Mellstrand-Navarro et al., 2014). Hormonal influences on bone density, occupational factors and differences in healthcare-seeking behaviour may further contribute (Ballering et al., 2023; Patel et al., 2013). Moreover, female patients might be treated less often by surgery than men, despite having similar or worse injuries (Pechlivanidou et al., 2023; Siccoli et al., 2018). Our findings reaffirm that osteotomies are largely procedures done in midlife and later adulthood. The tapering incidence in the elderly could reflect lack of fitness for surgery owing to comorbidities and also adherence to Swedish national guidelines advocating primary fixation in otherwise healthy older patients, which may reduce the need for later osteotomy (Mellstrand Navarro, 2021; Schmidt et al., 2022).

The marked dip in surgical volumes during 2020 highlights the vulnerability of elective surgical pathways to systemic disruptions. Sweden, like many countries, reallocated healthcare resources in response to the the pandemic, which temporarily reduced capacity for elective procedures. Similar declines have been described in other healthcare systems (Al-Jabir et al., 2020; Feier et al., 2022). Notably, no clear rebound effect was observed in the following years.

The variability in osteotomy incidence across Sweden raises concerns about equitable access to specialized hand surgery. Variations may stem from differences in referral pathways, surgeon distribution, local surgical culture and especially variations in incidence of primary operative treatment across the country (Saving et al., 2018). Moreover, proximity to specialized centres significantly influences the likelihood of undergoing surgery (Goldberg et al., 2014; Saving et al., 2018). Addressing these disparities will require deliberate policy measures, such as targeted redistribution of surgical expertise and structured regional collaborations with proven concepts such as telehealth consultations or virtual clinics (Davey et al., 2020; Johnson et al., 2025; Khan et al., 2020).

A continued decline in osteotomy procedures has major implications for surgical education. As case volumes decrease, exposure for trainees will inevitably diminish, potentially jeopardizing the transmission of technical expertise. This may result in a ‘generation gap’ where fewer surgeons are proficient in osteotomies, even though select patients will continue to benefit from the procedure. Similar concerns have been raised in the context of hip replacement and other high-skill orthopaedic interventions (Judge et al., 2006). This might prompt centralization of complex cases and cross-border collaborations to ensure adequate surgical competence. To maintain surgical proficiency across regions despite declining volumes, structured visiting consultant clinics with a mentorship programme could help preserve and teach local expertise while avoiding the dispersion of rare cases. In addition, rotating specialist services with scheduled outreach clinics and operative sessions in underserved areas may reduce regional disparities while ensuring access to high-quality care (Gruca et al., 2021).

The declining incidence may also reflect improvements in primary fracture fixation, early diagnosis and preventive strategies that reduce the likelihood of malunion or deformity requiring osteotomy, all highlighted in the Swedish national care programme for DRFs (Mellstrand Navarro, 2021; Schmidt et al., 2022). Earlier surgical fixation has the potential to further decrease the incidence of osteotomies, as delayed surgical fixation is associated with greater difficulties in anatomical reduction (Wadsten et al., 2025).

This study primarily provides ‘health system significance’ by quantifying national trends, demographic patterns and regional variation relevant to workforce planning, resource allocation and surgical education; however, the ‘clinical practice significance’ is inherently more limited, as registry data cannot determine appropriateness of indications, surgical technique, complications, functional outcomes or patient experience. Looking forward, several research areas warrant further study. First, linking registry data to functional outcomes would provide a greater understanding of whether declining osteotomy rates reflect improved disease prevention or unmet surgical need. Second, qualitative studies exploring patient and surgeon decision-making could illuminate the non-biological drivers of observed trends. Third, health economic analyses are warranted to evaluate if shifts away from osteotomy are cost-neutral. Finally, as predictive modelling suggests further decreasing trends of these procedures until 2040, continuous surveillance is essential to ensure that patients requiring surgery are not overlooked. Nevertheless, these models may overestimate future reductions in osteotomies; while improved primary fracture management could lower the incidence of osteotomies, congenital and acquired deformities will continue to necessitate surgical correction. Moreover, the decrease during the pandemic might increase the projected downward trends.

The strengths of this study lie in its comprehensive national coverage, long observation period and predictive modelling approach. Several limitations should be considered when interpreting the results of this study. First, the reliance on registry-based data introduces potential risks of coding errors and misclassification, as the accuracy of NOMESCO procedure codes depends on correct reporting by clinicians and hospitals. Although the NPR is well validated, minor discrepancies may still occur. Second, the registry does not provide detailed clinical information, such as radiographic severity, exact type of osteotomy, indication for osteotomy, functional impairment, comorbidities or patient-reported outcomes. These factors are important for understanding the clinical decision-making process and the true burden of disease but were not available for analysis. Third, while incidence rates were adjusted for population size, we were unable to account for changes in referral patterns, access to specialized centres or evolving surgical indications over time. These systemic factors may have contributed to the observed regional trends. Fourth, the analysis of regional differences was based on patients’ reported treatment location, but we could not assess inter-regional patient migration, e.g. individuals travelling to tertiary centres for surgery, which may have influenced regional incidence rates. Finally, the data were restricted to Sweden’s publicly reported registry; while the findings are highly generalizable within Sweden owing to universal healthcare, their external validity to other countries with different healthcare systems may be limited.

In conclusion, this nationwide study demonstrates a sustained decline in osteotomies of the wrist and hand in Sweden over a 16-year period, with predictive modelling indicating further reductions. Women consistently underwent more procedures than men, and most surgeries were performed in middle-aged and older adults. Regional differences highlight potential inequities in access to specialized surgical care. These findings indicate the need for ongoing surveillance of surgical trends, targeted strategies to address disparities and adaptation of surgical training to maintain expertise in low volume but clinically valuable procedures.
